# Proceedings from the 2025 Midwest Pediatric Device Consortium showcase featuring software as a medical device

**DOI:** 10.1186/s12919-026-00379-z

**Published:** 2026-05-11

**Authors:** Nina Echelmeyer, McKay Noll, Animesh Tandon, David M. Eckmann, Cory N. Criss

**Affiliations:** 1 The Midwest Pediatric Device Consortium, Nationwide Children’s Hospital, Columbus, OH 43215 USA; 2 Department of Pediatric Cardiology, Cleveland Clinic Childrens, Cleveland, OH 44195 USA; 3 Department of Anesthesiology, The Ohio State University Wexner Medical Center, Columbus, OH 43210 USA; 4 Center for Medical and Engineering Innovation, The Ohio State University, Columbus, OH 43210 USA; 5 Department of Surgery, Nationwide Children’s Hospital, Columbus, OH 43205 USA

**Keywords:** Software as a medical device, SaMD, Midwest pediatric device consortium, Innovation, Food and drug administration

## Abstract

On July 9, 2025, the Midwest Pediatric Device Consortium hosted its second showcase at the MidTown Collaboration Center in Cleveland, OH. This meeting convened clinicians, engineers, regulators, entrepreneurs, and policy stakeholders to discuss the challenges and opportunities in pediatric medical device development. The program included expert panel discussions addressing value demonstration in healthcare, data-driven post market surveillance, cybersecurity risk and data protection, artificial intelligence, clinical algorithms, and pediatric-specific considerations. This culminated in a pitch competition focused on Software as a Medical Device. This manuscript provides a descriptive summary of the meeting proceedings and synthesizes themes that emerged from panel discussions. The themes underscored the persistent structural barriers in pediatric device development, including alignment of value across stakeholders, limitations of real-world evidence infrastructure, data security challenges, and complexities in artificial intelligence driven technologies. These proceedings are intended to inform clinicians, innovators, and policymakers engaged in pediatric medical device development rather than serve as a formal program evaluation.

## Background: the Midwest Pediatric Device Consortium

The Midwest Pediatric Device Consortium (MPDC) is one of five US Food and Drug Administration (FDA) funded Pediatric Device Consortia established to support the development, regulatory advancement, and clinical adoption of pediatric medical devices. Founded in 2023, the MPDC operates as a multi-institutional collaboration among pediatric hospitals, academic institutions, and regional innovation organizations within Ohio. Participating institutions include Nationwide Children’s Hospital, The Ohio State University, Cincinnati Children’s Hospital, and Cleveland Clinic Children’s Hospital. These partners contribute complementary expertise in pediatric clinical care, engineering design, outcomes research, regulatory science, and technology commercialization. The MPDC also collaborates with regional innovation and investment organizations to support early prototyping, business development, and access to non-dilutive funding opportunities. This arrangement is designed to address known barriers in pediatric device innovation, including limited market incentives, fragmented expertise, and challenges in navigating clinical validation and trials, regulatory, and commercialization pathways. The MPDC employs a structured intake and review process to assess pediatric medical device concepts and companies. A centralized digital platform supports proposal evaluation, advisory matching, and tracking of project progress. Through this infrastructure, innovators are connected with clinical experts, engineers, regulatory specialists, and commercialization advisors based on project-specific needs. Since its inception, the MPDC has engaged more than 150 advisors and together reviewed project applications encompassing a wide array of pediatric clinical domains. Early outcomes associated with MPDC activities include progression of supported projects to feasibility testing, preclinical development, regulatory engagement, and federal small-business innovation research funding. More than 50 projects from across the country have received targeted technical, regulatory, or commercialization support, and over $1 million in pediatric device development funding has been awarded or facilitated since program initiation. In parallel, the MPDC has launched broader initiatives related to post-market safety, pediatric device registries, educational programming, and data-sharing collaborations.

### Showcase overview

The MPDC showcase was structured to highlight both ecosystem-level challenges and emerging solutions in pediatric device development. The agenda included expert panel discussions interspersed with startup pitch sessions focused on Software as a Medical Device (SaMD).

**Table:** 

**Showcase Agenda**	
Opening Remarks Panel 1: Demonstrating Value in Healthcare Start-Up Company Pitches (Group 1) Panel 2: Data-Driven Post-Market Surveillance Start-Up Company Pitches (Group 2) Panel 3: Protecting Sensitive Data in a Digital World Panel 4: From Algorithm to Application Panel 5: Let’s Talk Peds Closing Remarks	

### Panel 1: Demonstrating value in healthcare

The opening panel discussion featured professionals across the innovation lifecycle who shared their perspectives on what it takes to successfully bring a product into a hospital system. Panelists represented perspectives from hospital innovation programs, clinical care, and startup leadership. Discussion emphasized that value extends beyond clinical efficacy to include economic impact, scalability, workflow integration, and patient safety. Panelists noted that health care systems increasingly rely on a formal value analysis process, requiring innovators and champions to demonstrate measurable improvements in outcomes, efficiency, and costs. From a clinical perspective, usability, safety, and patient outcomes were identified as the obvious non-negotiable qualifications for adoption, especially in the neonatal and pediatric spaces. On top of these minimum requirements, the processes that value analysis teams utilize to evaluate prospective medical devices at each institution are highly variable and only partially standardized. Entrepreneurs are recommended to engage with value analysis committees early in the commercialization process.

Entrepreneurial perspectives highlighted the importance of understanding payer incentives and reimbursement strategies very early in development. Examples were provided to demonstrate early supply chain involvement and addressing reimbursement considerations are essential. Whether innovating in neonatology or maternal health, value in healthcare is multidimensional. It must be clinically meaningful, economically justified, logistically feasible, and emotionally trustworthy.

#### Key themes

Across the discussion, panelists emphasized that value in healthcare innovation is multidimensional. Successful adoption requires alignment among clinicians, administrators, payers, and patients, with clear articulation of clinical benefit, economic justification, and operational feasibility. Trust, incentive alignment, and stakeholder-specific communication were identified as critical factors in navigating health system adoption pathways.

### Panel 2: Data driven post market surveillance

This panel examined how advances in data infrastructure, artificial intelligence (AI), and real- world evidence (RWE) are reshaping post-market surveillance for medical devices, with particular attention to challenges relevant to pediatric populations. Panelists represented perspectives from regulatory science, health systems, and national data coordination initiatives.

Discussion highlighted a shift from traditional, reactive surveillance models toward more proactive approaches that leverage continuous data streams and real-world performance monitoring. Panelists noted that existing post-market surveillance frameworks were largely designed for static devices and may be poorly suited for software-based and AI-enabled technologies that evolve over time or encounter data distributions different from those used during development. Several speakers emphasized that device performance and safety cannot be interpreted without attention to clinical and operational context. Changes in staffing models, clinical workflows, patient mix, or data input quality may substantially influence real-world device behavior after deployment. As a result, surveillance systems that rely solely on outcome signals without capturing contextual variables risk misinterpreting safety or effectiveness trends. Pediatric-specific challenges were a central focus of the discussion. Panelists noted that small patient populations, rare conditions, and rapid developmental changes limit the usefulness of single-institution datasets. These constraints increase the importance of multi-institutional collaboration and data aggregation to improve signal detection and safety assessment for pediatric devices. Participants also underscored the need to ensure that surveillance efforts intentionally include pediatric populations, which are often underrepresented in post-market data sources. The panel further explored emerging collaboration models aimed at strengthening RWE infrastructure. Partnerships among manufacturers, academic medical centers, health systems, and commercial data providers were described as increasingly important for enabling scalable post- market studies. National initiatives designed to connect device developers with vetted data partners were discussed as potential mechanisms for reducing barriers to evidence generation while maintaining methodological rigor. Regulatory considerations were also addressed. Panelists observed that while regulators remain actively engaged in advancing post-market surveillance capabilities, existing regulatory pathways have not fully adapted to continuous or adaptive monitoring models. Ongoing discussions were noted regarding how active surveillance infrastructure might be incorporated into future regulatory frameworks and funding mechanisms.

#### Key themes

Across the discussion, panelists emphasized that effective post-market surveillance for modern medical devices requires continuous, context-aware data collection and multi-source RWE. Early engagement with data and regulatory partners, intentional inclusion of pediatric populations, and collaboration across institutions were identified as critical strategies for improving device safety and performance monitoring beyond initial approval.

### Panel 3: Protecting sensitive data in a digital world

The discussion of post-market surveillance naturally led into broader considerations of data governance and security. As device monitoring increasingly relies on continuous data collection, multi-institutional data sharing, and cloud-based analytics, panelists noted that the integrity, privacy, and security of these data streams become inseparable from patient safety. These considerations set the stage for the subsequent panel on protecting sensitive data in a digital healthcare environment. This panel focused on the increasing importance of data protection and cybersecurity in the development and deployment of digital health technologies and connected medical devices. Panelists brought perspectives from device development, health system cybersecurity, software architecture, and risk management, reflecting the multidisciplinary nature of cybersecurity challenges in healthcare. Discussion emphasized that cybersecurity is a foundational component of patient safety and system trust, rather than an optional technical feature. Panelists noted that early-stage companies and clinical innovators often underestimate both the scope and cost of cybersecurity requirements, particularly as devices become more interconnected and reliant on cloud-based infrastructure. Beyond device engineering, attention must be given to data privacy, interoperability with electronic health records, remote connectivity, and user authentication, each of which introduces potential vulnerabilities. Several speakers highlighted the complexity of modern healthcare environments as a key source of risk. Health systems typically operate heterogeneous information technology (IT) ecosystems with multiple legacy systems, third-party integrations, and delayed update cycles. As a result, vulnerabilities may arise not only from a device itself, but also from external vendors, subcontractors, or data partners. Panelists emphasized the importance of understanding data flows end-to-end and maintaining visibility into third-party dependencies throughout the product lifecycle. The panel also addressed the concept of shared responsibility in cybersecurity. While cloud service providers offer baseline security infrastructure, panelists cautioned that responsibility for application-level security, data governance, and regulatory compliance remains with device developers and deploying institutions. Contractual agreements were identified as an often- overlooked source of cyber risk, with panelists noting that warranties, indemnification clauses, and breach notification requirements can have significant operational and financial implications. Planning for failure was presented as a central principle of effective cybersecurity strategy. Panelists stressed that breaches and system failures should be assumed rather than treated as unlikely events. Early adoption of structured risk management frameworks, incorporation of security considerations into design controls, and development of incident response plans were discussed as essential practices. These approaches were framed as complementary to usability and safety engineering, rather than competing priorities. Pediatric considerations were also discussed. Panelists noted that pediatric data are particularly sensitive to security concerns due to their longitudinal nature and the involvement of minors, increasing the ethical and regulatory stakes of data protection failures. Trust from families and clinicians was described as especially fragile in pediatric settings, reinforcing the need for transparent data governance and proactive risk mitigation.

#### Key themes

Across the discussion, cybersecurity emerged as a core element of patient safety, regulatory compliance, and organizational trust in digital health. Panelists emphasized that effective data protection requires early planning, clear understanding of data flows and responsibilities, and integration of security considerations throughout the device lifecycle. As digital and AI-enabled medical technologies continue to expand, proactive cybersecurity strategies were identified as essential to sustaining innovation and protecting patients.

### Panel 4: From algorithm to application

This panel focused on the practical challenges of translating algorithms and software innovations into clinically deployed medical devices. Panelists represented expertise in software development, AI, and medical device commercialization, offering perspectives on balancing rapid innovation with regulatory, operational, and safety requirements.

Discussion emphasized the importance of disciplined product development in regulated healthcare environments. Panelists noted that while AI and modern software tools have lowered technical barriers to prototyping, successful translation requires careful prioritization of core functionality. Early-stage teams were encouraged to focus on a limited set of features that address clearly defined clinical needs rather than pursuing broad or complex feature sets that may complicate validation and regulatory review.

A central theme was the critical role of workflow and user-centered design. Panelists highlighted that healthcare systems operate with diverse clinical workflows, IT constraints, and stakeholder roles. Failure to understand these workflows early can lead to usability challenges, safety risks, and barriers to adoption. Mapping interactions across multiple personas—including clinicians, patients, caregivers, IT teams, and administrators, was identified as essential for aligning product design with real-world clinical environments. Existing regulatory guidance, such as human factors and usability frameworks, was discussed as a resource for structuring this work. Security considerations were described as increasingly intertwined with algorithm development and deployment. Integration of AI, cloud-based services, and interoperable data standards introduces new risk surfaces that extend beyond traditional device boundaries. Panelists emphasized the importance of understanding what data are accessed, stored, processed, or shared by AI-enabled systems and of establishing governance mechanisms to manage these risks. Cost monitoring and resource utilization associated with AI deployments were also identified as emerging operational considerations. Regulatory dynamics were another focus of the discussion. Panelists observed that regulatory review processes for software-based medical devices are evolving rapidly, with increasing expectations for documentation, transparency, and lifecycle management. The potential use of automated tools to support regulatory review was discussed as a factor that may accelerate feedback timelines while simultaneously increasing scrutiny. As a result, panelists emphasized the importance of embedding regulatory and quality considerations into product development from the earliest stages.

#### Key themes

The discussion underscored that successful translation from algorithm to application depends on early attention to clinical workflows, security, and regulatory requirements. While AI can accelerate development, sustainable deployment requires disciplined design, user engagement, and proactive risk management throughout the product lifecycle.

### Panel 5: let’s talk peds

The final panel addressed pediatric-specific considerations in medical device innovation and the operational realities of sustaining pediatric-focused development efforts. Panelists discussed both the clinical distinctiveness of pediatric populations and the structural challenges associated with funding, regulation, and commercialization in this space.

Discussion highlighted that pediatric medical device development is constrained by small market sizes, heterogeneous patient populations, and limited availability of venture capital. Panelists noted that pediatric-specific startups receive a disproportionately small share of private investment, creating reliance on non-dilutive funding sources and public–private partnerships to support early- stage development. Regulatory funding caps and administrative requirements were described as additional constraints that shape programmatic decision-making. Operational considerations were a major focus of the discussion. Panelists described the substantial administrative, legal, and compliance work required to support pediatric device development, much of which is not visible to external stakeholders. These activities were framed as essential to maintaining regulatory compliance, financial accountability, and long-term program sustainability. The importance of coordinated governance across institutions was emphasized as a means of reducing redundancy and improving access to shared infrastructure. The panel also addressed strategic approaches to mitigating pediatric market challenges. Emphasis was placed on de-risking early-stage projects through access to clinical champions, pilot opportunities, as well as targeted technical and regulatory support. Panelists discussed the value of distributed consortium models that prioritize collaboration over competition and facilitate cross- institutional access to expertise and resources.

#### Call to action

Panelists concluded that addressing persistent gaps in pediatric medical device development will require coordinated action across clinical, regulatory, funding, and policy domains. They emphasized the need for continued investment in shared infrastructure, expanded access to non- dilutive funding mechanisms, and intentional inclusion of pediatric populations in post-market data systems. Strengthening cross-institutional collaboration, aligning incentives across stakeholders, and generating transparent evidence of impact were identified as priorities for advancing pediatric medical technologies from concept to clinical use.

#### Pitch competition highlights

The MPDC showcase included a pitch competition featuring eight MPDC-engaged pediatric medical device companies, with a focus on software-based medical technologies. The participating companies reflected a diverse range of pediatric clinical applications, including neonatal care, pain management, cardiology, metabolic disorders, neurology, sleep medicine, and oncology. Each company presented its innovation to an audience comprising clinicians, health system representatives, service providers, and investors. Awards up to $30,000 were allocated based on audience ranking. Rekovar presented a neonatal intensive care monitoring platform that integrates wearable, non- invasive sensors with audio and video monitoring and AI–based analytics to support automated symptom scoring and clinical decision-making. The system is designed to reduce clinician burden and support transitions from inpatient to home-based care for neonatal conditions. AngelEye Healthcare described efforts to expand its neonatal monitoring ecosystem through AI- enabled video analysis and pressure-sensitive sensing technologies aimed at detecting clinically relevant movement and neurological patterns in infants within the neonatal intensive care unit environment. OpalGenix presented a software-based decision support platform that incorporates pharmacogenetic data, clinical risk factors, and AI algorithms to guide personalized opioid prescribing across pediatric and adult populations, including postoperative care and neonatal abstinence syndrome risk assessment.

Atrility Medical described a device that integrates with surgical pacing wires to provide high- fidelity atrial waveform data, supporting improved arrhythmia detection without disrupting existing workflows. The company outlined a transition toward a software and AI-enabled platform to support real-time rhythm classification and predictive analytics. Kilele Health presented a wearable biosensor platform based on aptamer technology for continuous, real-time measurement of biochemical markers. The platform is initially focused on phenylketonuria, with planned expansion to additional pediatric and adult conditions requiring longitudinal biomarker monitoring. The team from NEOMED described a virtual reality based concussion detection platform that combines immersive testing with sensor data capture and machine learning–based diagnostics. The system is designed to provide objective concussion assessment without requiring baseline testing. Seluna Ltd presented a machine learning–based software platform for automated analysis of sleep study data to support diagnosis, severity classification, and treatment planning for pediatric sleep apnea and related conditions.

GRAIMED described an AI–driven decision support platform integrating imaging and genomic data to improve diagnostic accuracy and reduce invasive procedures in pediatric oncology. Potential applications include surgical planning and clinical trial matching. Collectively, the pitch competition highlighted prevailing trends in pediatric medical device innovation, including the increasing prominence of software-based diagnostics, AI–enabled monitoring, and platform technologies designed to integrate into existing clinical workflows and care environments.

Following the pitch presentations, audience members ranked the companies based on perceived readiness and impact. Awardees included Kilele Health (first place, $30,000), Atrility Medical (second place, $20,000), OpalGenix (third place, $10,000), and GRAIMED (fourth place, $7,500). In addition to direct financial awards, the MPDC described plans to provide structured post- showcase support to participating companies through an affiliated accelerator framework. Companies not receiving monetary awards were offered tailored advisory and technical assistance focused on regulatory strategy, clinical validation planning, reimbursement considerations, and commercialization readiness. This support will leverage MPDC’s existing advisory network and partner organizations to provide time-limited, milestone-driven engagement aimed at addressing gaps identified during the evaluation process. Panelists emphasized that this approach is intended to extend the impact of the showcase beyond one-time funding decisions by supporting continued project maturation and increasing readiness for downstream investment, regulatory engagement, or clinical adoption.

#### Impact and engagement

MPDC engagement reflects substantial participation from pediatric medical device innovators, regional industry partners, and commercialization stakeholders. Since inception, the MPDC reviewed more than 330 project submissions and processed over 110 applicants through its funding and evaluation mechanisms. Utilizing its centralized deal-flow database and matchmaking infrastructure, MPDC facilitated coordinated engagement among approximately 80 companies and 150 advisors spanning regulatory, engineering, clinical, and business expertise. More than 50 projects have received individualized technical, regulatory, or commercialization support, and over $1 million in funding has been deployed or facilitated to advance pediatric device development by the MPDC from its inception through the end of 2025. Early translational indicators include progression of supported projects to feasibility testing, advancement of small- business grant proposals, and early-stage regulatory interactions. Regional symposia and educational convenings have served as catalysts for cross-institutional collaboration, connecting device developers with clinical champions, translational engineers, and potential investment partners. Collectively, these activities have broadened engagement with pediatric device innovation in the Midwest and enhanced the capacity of startups and clinical innovators to access expertise critical to advancing pediatric medical technologies.

## Data Availability

Access to the original recordings is subject to review of a written request to the corresponding author.

## Abbreviations

AI: Artificial intelligence
CEO: Chief Executive Officer
CFO: Chief Finance Officer
CMO: Chief Marketing Officer
CNN: Cable News Network
COO: Chief Operating Officer
FDA: Food and Drug Administration
IT: Information Technology
MD: Doctor of Medicine
MPDC: Midwest Pediatric Device Consortium
NESTcc: National Evaluation System for health Technology Coordinating Center
RWE: Real-world evidence
VP: Vice President
VR: Virtual reality

